# High-Capacity Reversible Hydrogen Storage on Calcium-Decorated Silicene: A DFT Study

**DOI:** 10.3390/nano16171060

**Published:** 2026-08-26

**Authors:** Na Xiong, Ying Zhang, Lun Tan, Gui Lei, Shulin Yang, Zhigao Lan

**Affiliations:** 1 School of Physics and Electronic Information, Huanggang Normal University, Huanggang 438000, China; 2 Faculty of Physics and Electronic Sciences, Hubei University, Wuhan 430062, China

**Keywords:** Ca-decorated silicene, DFT, hydrogen storage, Kubas-like interaction

## Abstract

First-principles DFT calculations have been employed to examine the hydrogen storage behavior of Ca-decorated silicene. A single Ca atom binds to the hollow site of the silicene monolayer with a binding energy of −2.348 eV, the magnitude of which exceeds the cohesive energy of bulk Ca, thus preventing Ca clustering. Pristine silicene interacts weakly with H_2_, with an adsorption energy of only −0.047 eV, while Ca decoration notably enhances the binding affinity to −0.561 eV. Each Ca site can accommodate up to five H_2_ molecules through a Kubas-like interaction, with an average adsorption energy of −0.327 eV. With Ca atoms decorating both sides of the silicene, the system delivers a hydrogen storage capacity of 7.5 wt% and complete H_2_ release at approximately 380 K as revealed by AIMD simulations. Our findings suggest that Ca-decorated silicene could serve as a viable material for reversible hydrogen storage applications.

## 1. Introduction

The transition toward a sustainable energy economy has placed hydrogen gas (H_2_) in a central role, yet its widespread utilization remains constrained by the lack of efficient storage technologies [1,2,3]. Among the various strategies explored, solid-state storage in lightweight materials offers a possible pathway to meet the U.S. Department of Energy (DOE) target of 5.5 wt% for on-board systems [2,4]. Metal hydrides, such as perovskite-type LiCrH_3_ and KCrH_2_, provide hydrogen storage densities of 4.38 wt% and 3.11 wt%, respectively, yet their H_2_ release typically requires high temperatures [5]. Porous materials and carbon-based nanostructures have likewise shown improved H_2_ uptake when functionalized with metal atoms. For instance, Sc-decorated porous B_40_ fullerene stores 6.18 wt% H_2_ in its bulk form [6], while Y-decorated C_24_ fullerene reaches 8.84 wt% and the Sc_20_C_60_H_70_ cluster attains 4.20 wt% [7,8]. Beyond these systems, two-dimensional (2D) materials have gained increasing interest for hydrogen storage, benefiting from their large surface areas and chemically versatile surfaces [9,10,11]. Nevertheless, pristine 2D sheets generally bind H_2_ too weakly, requiring surface modification to strengthen the binding affinity [10,12]. Decoration with metal atoms has proven effective in this regard. For example, Na-decorated SiGe monolayers achieve a hydrogen storage capacity of ~7.79 wt% [13]. Similarly, Li-decorated α-C_3_N_2_, Li-modified C_4_N, Na-modified B_5_N_4_, and Na-decorated B_5_N_3_ monolayers exhibit capacities of 5.70 wt%, 8.0 wt%, 6.44 wt%, and 7.80 wt%, respectively [14,15,16,17]. These examples illustrate that metal-functionalized 2D materials hold promise as hydrogen storage substrates.

Silicene, a silicon-based cousin of graphene, features a non-planar, buckled honeycomb lattice [18,19]. This puckered configuration, arising from reduced *π*-orbital overlap between adjacent Si atoms relative to planar graphene, grants silicene a larger lattice constant, enhanced chemical reactivity, and a tunable band gap [20,21]. These characteristics make silicene attractive for gas adsorption, as evidenced by prior studies on CO, NO_2_, NH_3_, and SO_2_ capture using silicene-based materials [22,23,24]. For hydrogen storage specifically, the buckled structure and partial *sp*^3^ character of silicene facilitate exohedral functionalization, suggesting that metal decoration could improve H_2_ uptake. Earlier DFT investigations have shown that Li-functionalized silicene reaches a storage capacity of 6.35 wt% with H_2_ adsorption energies of −0.17 eV/H_2_ [25]. Ni-decorated silicene, meanwhile, interacts with H_2_ through a Kubas-type interaction, exhibiting an adsorption energy of −0.439 eV [26]. Systematic studies of alkali and alkaline-earth metal decoration have identified K and Li as effective candidates, whereas Mg binds only weakly to silicene [27]. These observations underscore the critical role of the decorating species in governing the hydrogen storage performance of silicene-based systems.

In selecting a suitable decorating metal, calcium (Ca) presents several favorable attributes. Its low atomic mass minimizes the penalty to the gravimetric capacity, while its low ionization energy facilitates charge transfer to the substrate [28,29]. More importantly, the Ca-H_2_ interaction is primarily electrostatic: upon electron donation to the host, the resulting Ca^2+^-like cation generates a local electric field that polarizes the H-H bond, yielding adsorption strengths that are neither too weak for ambient retention nor too strong for subsequent release. This binding behavior differs from the d-orbital-mediated interactions typical of transition metals, where H_2_ dissociation and irreversible chemisorption may take place [30]. The viability of Ca as a hydrogen storage promoter has been demonstrated across various 2D platforms. For instance, Ao et al. found that Ca-decorated graphene with topological defects captures six H_2_ per Ca, with binding energies of −0.33 eV/H_2_ and a double-sided capacity of 5.2 wt% [28]. Likewise, Wang et al. reported that Ca-functionalized BN nanosheets with B vacancies bind six H_2_ per Ca at ~0.20 eV/H_2_, affording storage capacities of 7.6 wt% [29]. More recently, the study on Ca-decorated Irida-graphene revealed that Ca atoms are firmly anchored to eight-membered carbon rings, enabling the storage of 32 H_2_ molecules with a capacity of 8.0 wt% [31]. Even zero-dimensional systems, such as Ca-coated C_60_ fullerenes, have been predicted to achieve 6.2 wt% storage with up to 62 H_2_ molecules accommodated [32]. Encouragingly, these collective findings point toward the possibility that Ca-decorated silicene may also exhibit promising hydrogen storage behavior, yet few systematic investigations of this specific system have been reported.

In the present study, we perform a comprehensive first-principles DFT investigation of the hydrogen storage properties of Ca-decorated silicene. We first determine the most stable binding configuration for a single Ca adatom on the silicene surface and assess the thermodynamic preference for isolated atom dispersion relative to cluster formation. The stepwise addition of H_2_ molecules at the Ca site is then investigated to identify the saturation capacity consistent with the reversible adsorption energy criterion. To understand the electronic origin of the interaction, charge density difference, Mulliken charge analysis, and partial density of states are employed. In addition, molecular dynamics simulations are performed to assess the thermal stability and H_2_ desorption behavior of the decorated system at finite temperatures. This work aims to evaluate the potential of Ca-decorated silicene as a possible hydrogen storage medium and to offer theoretical insights for the design of silicon-based nanomaterials for energy applications.

## 2. Computational Methods

All DFT calculations reported herein were conducted with the CASTEP package as implemented in Materials Studio (MS) 8.0. The silicene monolayer was represented using a 4 × 4 supercell built from 32 Si atoms, with a vacuum layer of 25 Å applied along the surface normal to avoid spurious coupling between repeated slabs. The exchange-correlation energy was described by the Perdew-Burke–Ernzerhof functional in the generalized gradient approximation [11]. Since standard DFT fails to capture the dispersion forces that dominate H_2_ physisorption, we incorporated the DFT-D2 empirical correction of Grimme in all geometry optimizations and energy evaluations [33]. Ion-electron interactions were described using ultrasoft pseudopotentials. A cutoff energy of 600 eV was chosen after convergence testing, ensuring that the total energy variation remained within 1 meV per atom. We employed a 5 × 5 × 1 Monkhorst–Pack grid for structural relaxations and a finer 6 × 6 × 1 grid was applied for electronic structure calculations. Structural relaxations continued until the total energy change per atom was less than 1.0 × 10^−5^ eV, while the maximum forces, stress components, and atomic displacements were constrained to values below 0.03 eV/Å, 0.05 GPa, and 1.0 × 10^−3^ Å, respectively.

To confirm the dynamical stability of the relaxed structures, we computed phonon spectra using the finite displacement method within CASTEP [34], employing the nondiagonal supercell scheme of Lloyd-Williams and Monserrat [35]. The presence of ultrasoft pseudopotentials in our calculations makes this approach necessary, since the finite displacement technique is the only option for phonon calculations with these settings [34,36]. A displacement of 0.02 Å was used to construct the force constant matrices. The real-space force constants were then Fourier-transformed to obtain the dynamical matrices, which were subsequently diagonalized to yield the phonon frequencies [37].

Alongside these static calculations, we performed ab initio molecular dynamics (AIMD) simulations with the CASTEP code to evaluate the thermal stability and H_2_ desorption characteristics of the decorated systems [34]. The atomic forces were computed on the fly from the electronic structure within the DFT framework during these simulations [38]. All AIMD runs were conducted under the NVT ensemble using the Nosé–Hoover thermostat, with a total simulation time of 5 ps and a time step of 1.0 fs [39]. For the H-H stretching modes of the adsorbed H_2_ molecules, the experimental fundamental frequency of free H_2_ is 4161 cm^−1^ [40], which corresponds to roughly 125 THz and a vibrational period of about 8 fs. The selected 1 fs step ensures sufficient sampling of these rapid motions and is in line with typical values adopted in AIMD simulations of hydrogen-bearing systems [39,41]. All interatomic interactions were treated at the DFT level without employing any classical force field.

The adsorption stability of a Ca atom on silicene was quantified by the binding energy (*E_b_*_−*Ca*_):

*E_b_*_−*Ca*_ = *E_Ca_*_+*silicene*_ − *E_silicene_* − *E_Ca_* (1)

where *E_Ca_*_+*silicene*_, *E_silicene_*, and *E_Ca_* are the total energies of the Ca-decorated system, the pristine silicene monolayer, and an isolated Ca atom, respectively [2]. Negative values of *E_b_*_−*Ca*_ indicate exothermic decoration.

For hydrogen adsorption, the average adsorption energy per H_2_ molecule (*E_ads_*_−*av*_) for a system carrying *n* H_2_ was evaluated as:

*E_ads_*_−*av*_ = [*E_Ca_*_+*silicene+nH*2_ − *E_Ca_*_+*silicene*_ − *n*
*E_H_*_2_]/*n* (2)

and the sequential adsorption energy (*E_ads_*_−*n*_) associated with attaching the *n*-th H_2_ was obtained from:

*E_ads_*_−*n*_ = *E_Ca_*_+*silicene*+*nH*2_ − *E_Ca_*_+*silicene+*(*n*−1)*H*2_ − *E_H_*_2_ (3)

In these expressions, *E_Ca_*_+*silicene*+*nH*2_ or *E_Ca_*_+*silicene*+(*n−*1)*H*2_ denotes the total energy of the substrate with *n* or *n*−1 adsorbed H_2_ molecules, and *E_H_*_2_ is the energy of a free H_2_ molecule [27].

To estimate the temperature (*T_d_*) at which the stored H_2_ molecules can be released, we employed the Van ’t Hoff equation under standard pressure (*P/P*_0_ = 1):

*T_d_* = |*E_ads_*_−*av*_| × *k_B_*^−1^ × [Δ*S*/*R* − ln(*P/P*_0_)]^−1^ (4)

where |*E_ads_*_−*av*_| is the absolute value of the average adsorption energy per H_2_, *k_B_* is the Boltzmann constant (1.38 × 10^−23^ J/K), Δ*S* is the entropy change in H_2_ upon desorption (taken as 75.44 J·mol^−1^·K^−1^ for the transition from gas to liquid phase), and *R* is the universal gas constant (8.314 J·mol^−1^·K^−1^) [42].

## 3. Results and Discussion

### 3.1. Optimized Structure of the Silicene Monolayer and Ca-Decorated Silicene

**Figure 1:**
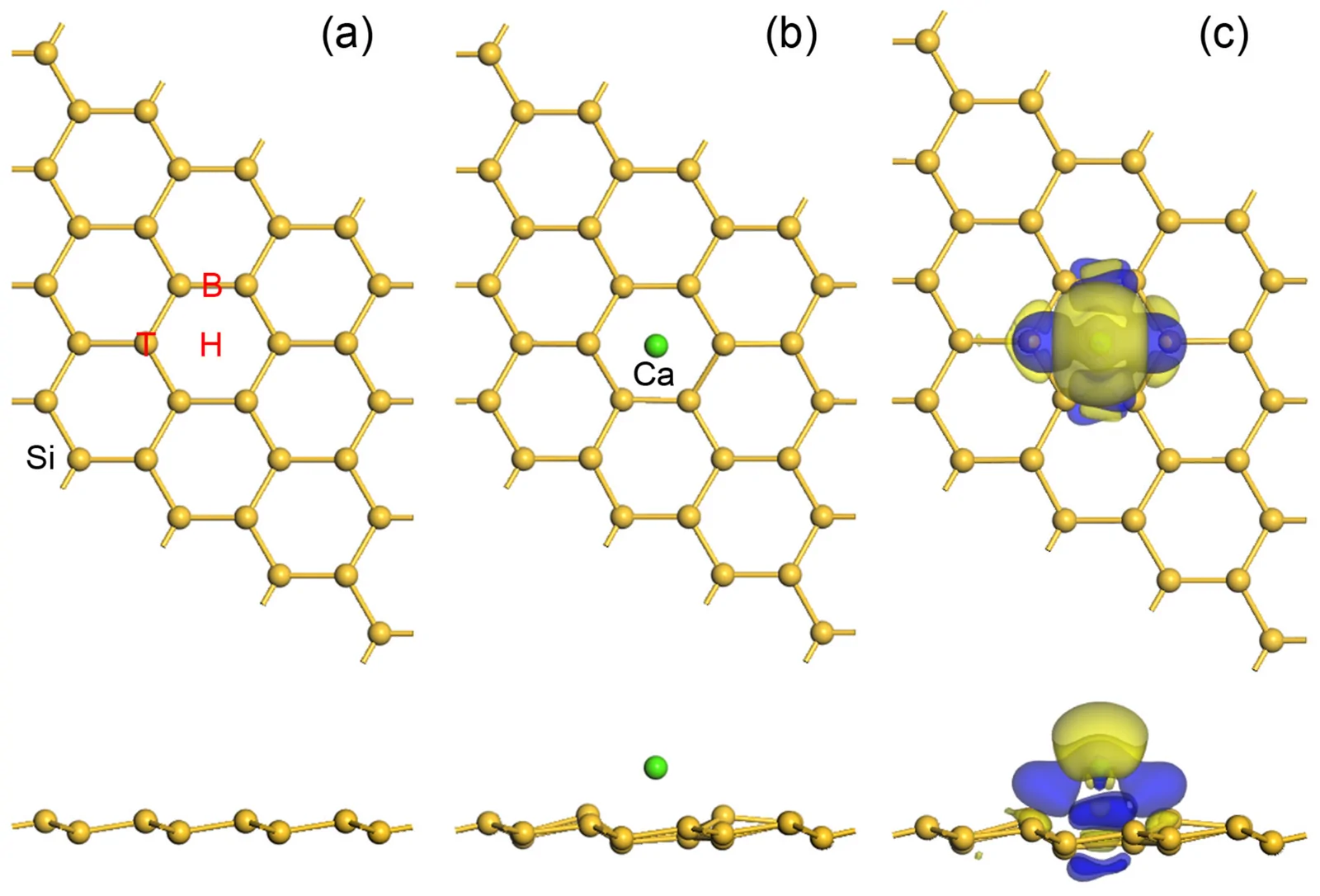
Top and side views of the fully relaxed pristine silicene (**a**), silicene decorated with one Ca atom, denoted as 1Ca-silicene (**b**), and (**c**) EDD map with isosurface value of 0.015 e/Å^3^. In (**c**), charge accumulation and depletion are represented by blue and yellow, respectively.

**Figure 2:**
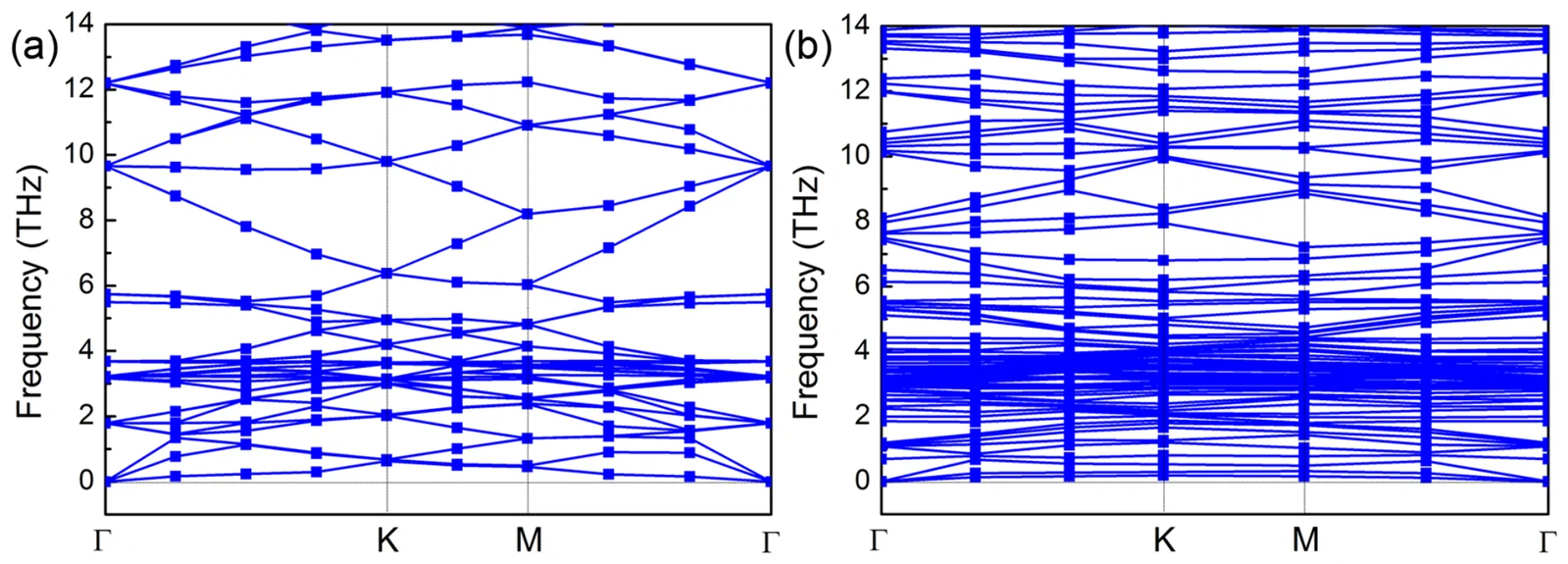
Phonon dispersion curves for pristine silicene (**a**) and 1Ca-silicene (**b**), with no negative modes detected.

The optimized silicene monolayer (Figure 1a) adopts a graphene-like buckled honeycomb configuration. The calculated Si-Si bond length is 2.267 Å, consistent with earlier theoretical values reported for silicene [24,25]. Unlike planar graphene, the Si atoms in the optimized silicene form two separate atomic layers, giving rise to a buckling height of 0.431 Å. The phonon dispersion in Figure 2a has no imaginary frequencies, indicating the dynamical stability of the pristine silicene monolayer [43].

To identify the most stable site for anchoring a single Ca atom on the silicene surface, three high-symmetry positions were examined, including the top site T located directly above a Si atom, the bridge site B positioned at the midpoint of a Si-Si bond, and the hollow site H at the center of a six-membered silicon ring (labeled in Figure 1a). The calculated *E_b_*_−*Ca*_ values for the T, B, and H sites are −1.729 eV, −1.865 eV, and −2.348 eV (Table 1), respectively, indicating that the hollow site is energetically preferred [42]. In the lowest-energy configuration of 1Ca-silicene, the Ca atom is positioned 1.816 Å above the hollow site (Figure 1b). The magnitude of |*E_b_*_−*Ca*_*|* at the H site exceeds the cohesive energy of bulk Ca, which is 1.84 eV per atom [44]. This indicates that isolated Ca atoms tend to disperse uniformly on the silicene surface rather than forming clusters [44]. To further evaluate the kinetic stability of Ca adatoms against clustering, we computed the diffusion barrier for a single Ca atom migrating across the silicene surface using the NEB method within CASTEP [34]. The initial and final configurations were taken as two neighboring hollow sites [39]. As shown in Figure S1, the calculated diffusion barrier is 1.451 eV, considerably larger than the room-temperature thermal energy of about 0.026 eV [45]. These results indicate that Ca atoms are kinetically resistant to surface migration and cluster formation under ambient conditions, which is consistent with the thermodynamic picture derived from the binding energy analysis [46]. The phonon spectrum of 1Ca-silicene (Figure 2b) also contains no imaginary frequencies, confirming its robust dynamical stability [43].

**Figure 3:**
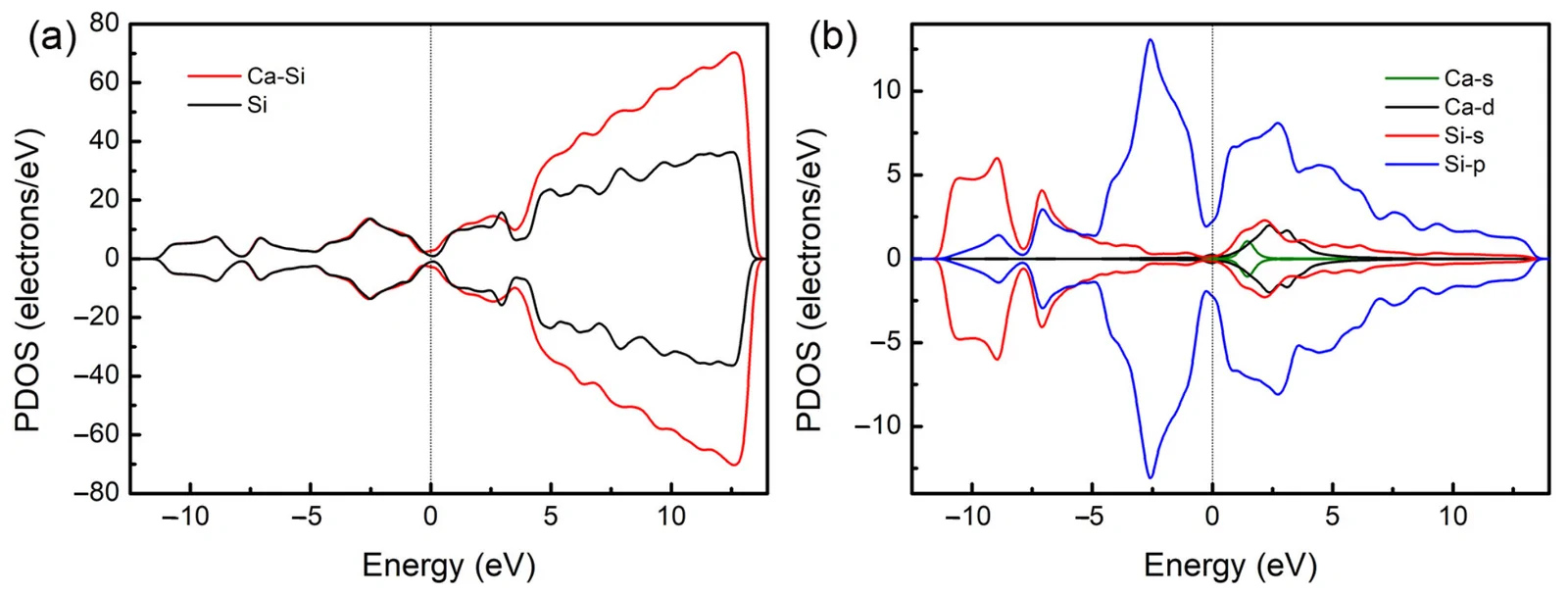
Comparison of DOS between pristine silicene and 1Ca-silicene (**a**), and PDOS highlighting the Ca-Si orbital coupling (**b**).

The strong adhesion of Ca to the silicene surface is accompanied by substantial charge redistribution. Electron density difference (EDD) analysis reveals that approximately 1.16 |e| is transferred from the adsorbed Ca atom to the silicene substrate (Figure 1c). This charge transfer generates a pronounced electrostatic attraction that firmly binds the Ca adatom to the surface [47]. Density of states (DOS) calculations (Figure 3a) further support this picture, showing that the electronic peaks of the 1Ca-silicene system shift toward higher energies relative to pristine silicene, indicative of a strong Ca-Si interaction [48]. As seen in Figure 3b, the partial density of states (PDOS) reveals substantial hybridization of the Ca *s/d* states with the Si *s*/*p* states from +0.366 eV to +5.245 eV, providing additional evidence for the robust electronic coupling between the adatom and the silicene [49]. These observations suggest that the Ca atom should be stably anchored at the hollow site of the silicene monolayer through a combination of strong electrostatic attraction and orbital hybridization.

### 3.2. Adsorption of One H_2_ on Pristine Silicene and 1Ca-Silicene

**Figure 4:**
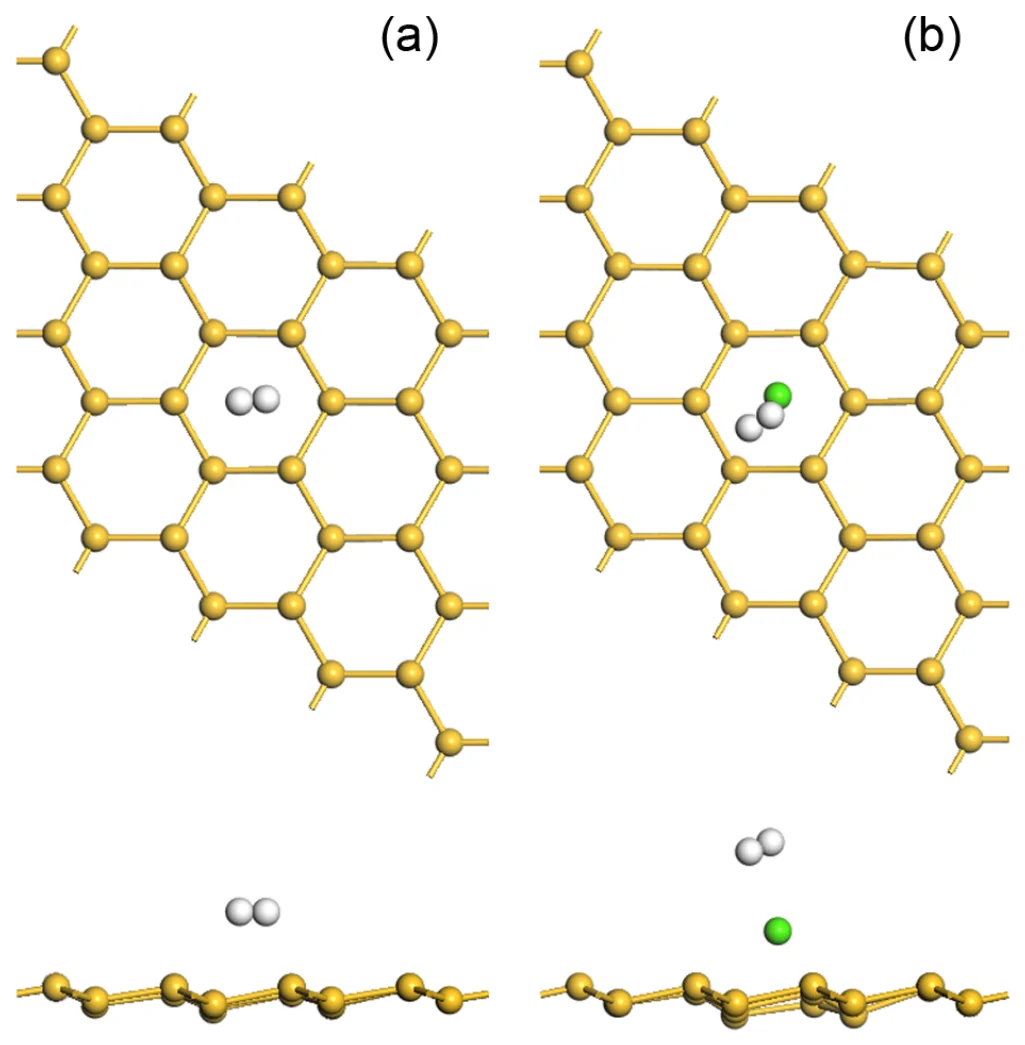
Optimized geometries for one H_2_ molecule adsorbed on (**a**) pristine silicene and (**b**) 1Ca-silicene.

The intrinsic hydrogen affinity of pristine silicene was evaluated by placing one H_2_ molecule at each of the three adsorption sites described in the previous section, yielding configurations denoted as 1H-Si-T, 1H-Si-B, and 1H-Si-H. Adsorption energies of −0.026 eV, −0.018 eV, and −0.047 eV were obtained for the T, B, and H sites, respectively, with the hollow site being energetically preferred. As shown in Figure 4a, the H-H bond length is 0.755 Å, which is nearly the same as that of free H_2_ (0.754 Å) [50], and the separations between H_2_ and the neighboring Si atoms lie between 3.601 Å and 4.119 Å. These observations indicate that H_2_ interacts only weakly with pristine silicene, owing to the scarcity of active sites on the clean surface [6,8]. Similar behavior has been documented for other pristine 2D sheets, including o-B_2_N_2_, B_4_C_3_, and GeC_5_ monolayers [10,33,51].

In contrast, when H_2_ was introduced onto 1Ca-silicene (denoted as 1H-1Ca-silicene), a distinctly different behavior was observed. As illustrated in Figure 4b, the molecule approaches the Ca site much more closely, with an adsorption distance of 2.261 Å, and the H-H bond stretches to 0.768 Å. The resulting adsorption energy is −0.561 eV, considerably more negative than the value for pristine silicene. This substantial difference suggests that Ca modification markedly enhances the H_2_ binding affinity of the silicene substrate [42]. Comparable improvements in hydrogen uptake have also been documented for other metal-functionalized two-dimensional systems, including Li-decorated SiB and Ca-decorated nanoporous CN monolayers [42,52]. To validate the reliability of the DFT-D2 dispersion correction, we performed additional benchmark calculations on the same 1H-1Ca-silicene configuration using the Tkatchenko–Scheffler (TS) dispersion correction scheme [53]. This scheme yields an adsorption energy of −0.557 eV, in excellent agreement with the DFT-D2 result. The deviation of only 0.004 eV reveals that the DFT-D2 results are robust and that the choice of dispersion correction does not appreciably affect the conclusions of this work. In addition, we also examined the dynamical stability of the hydrogenated system by computing the phonon spectrum for the 1H-1Ca-silicene configuration. The resulting phonon dispersion, displayed in Figure S2, shows no soft modes across the whole Brillouin zone, confirming that the structure remains stable upon H_2_ adsorption [43].

**Figure 5:**
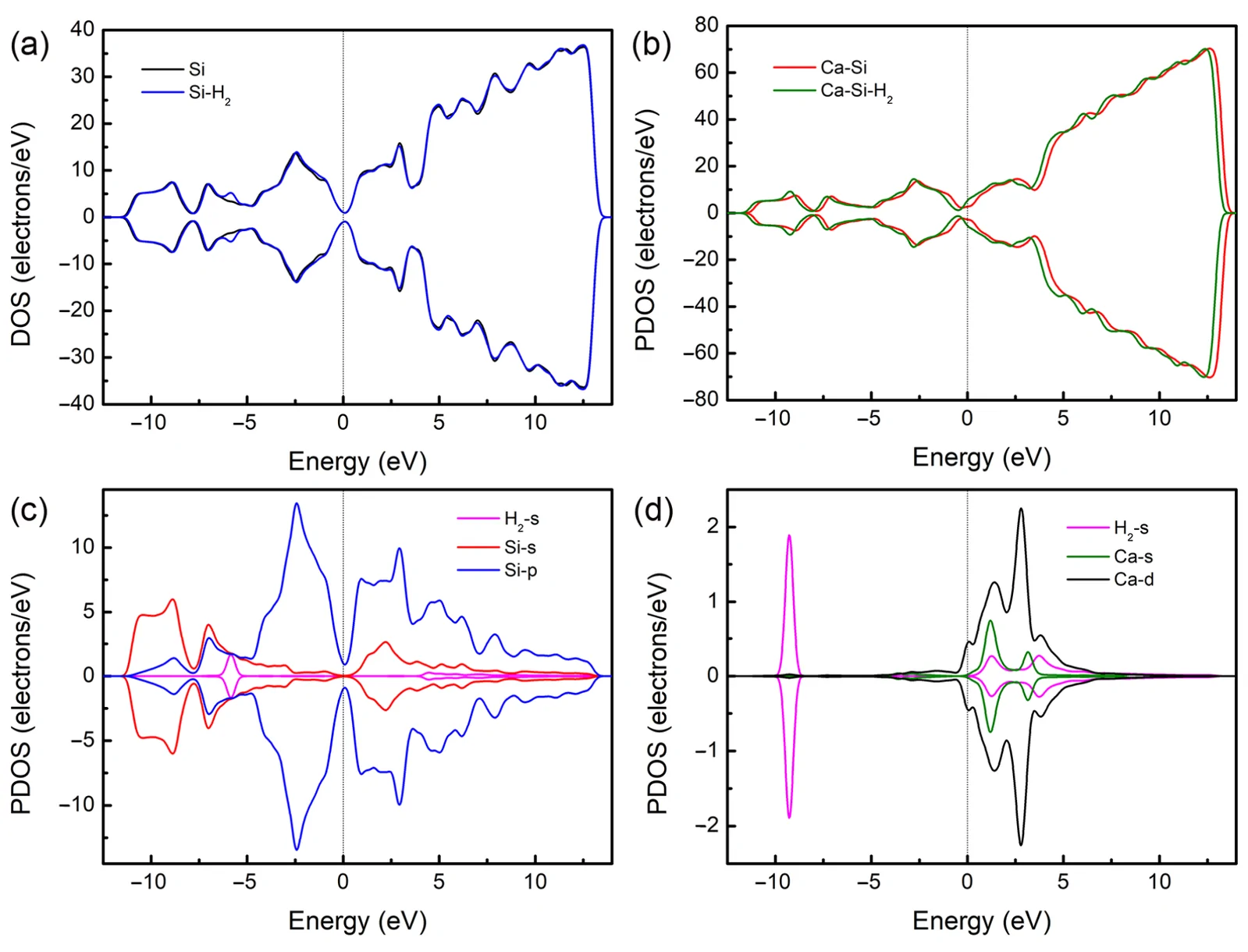
DOS for one H_2_ on (**a**) pristine silicene and (**b**) 1Ca-silicene, and the corresponding PDOS depicting orbital interactions on (**c**) pristine silicene and (**d**) 1Ca-silicene.

**Figure 6:**
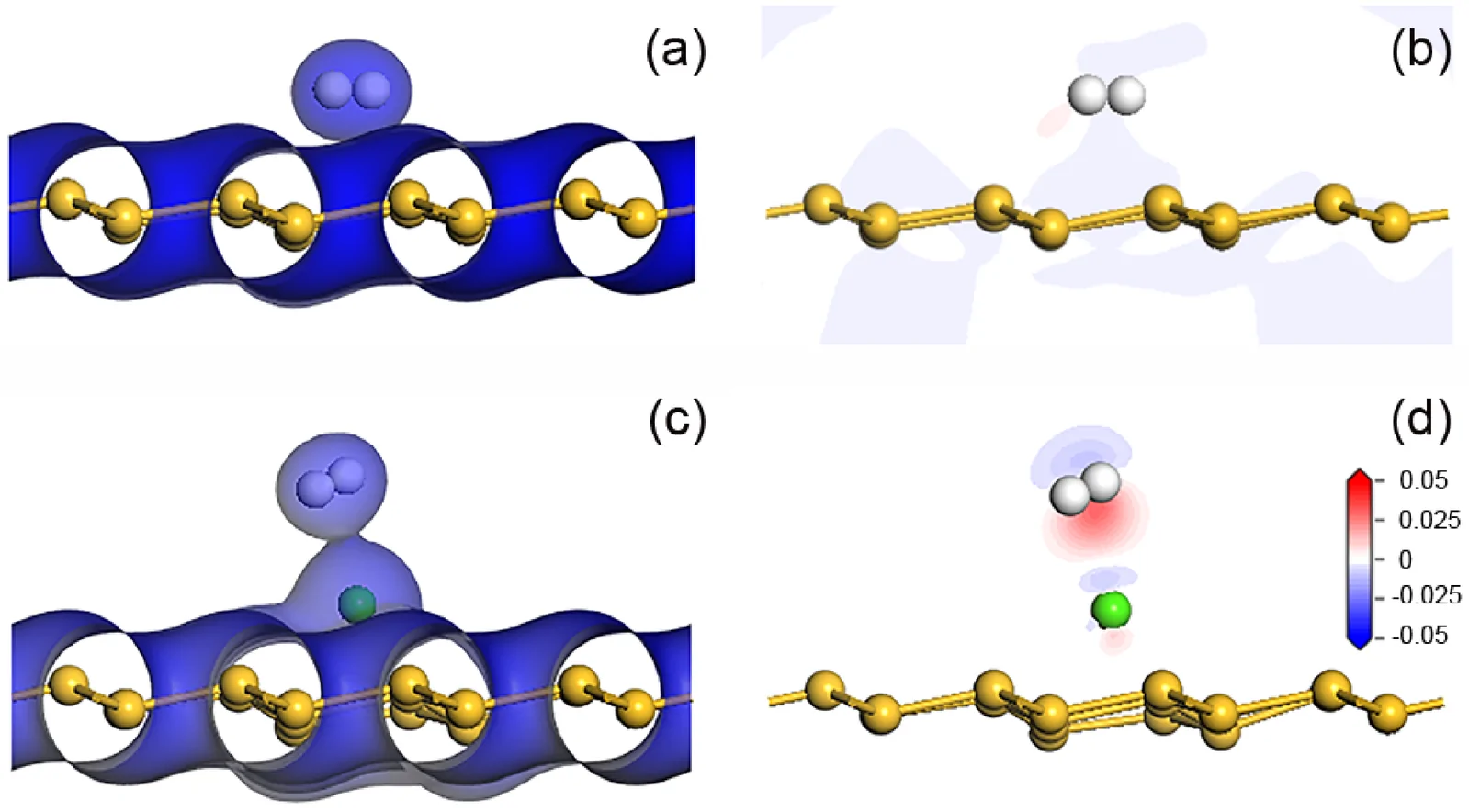
TED and EDD slice plots for one H_2_ adsorbed on (**a**,**b**) pristine silicene and (**c**,**d**) 1Ca-silicene. The color scale for the EDD slices in (**b**,**d**) ranges from −0.05 to +0.05 e/Å^3^, with red and blue representing charge accumulation and depletion, respectively.

To further understand the mechanism underlying the enhanced hydrogen adsorption performance of 1Ca-silicene, we performed DOS, PDOS, total electron density (TED), and EDD analyses for both adsorption systems. The DOS analysis in Figure 5a indicates that the electronic structure of pristine silicene remains essentially unaltered upon H_2_ adsorption, implying a negligible H_2_-silicene interaction [44]. This weak interaction is further confirmed by the minimal overlap in PDOS (Figure 5c) and TED plots (Figure 6a), consistent with the low adsorption energy.

For the Ca-decorated system, however, the DOS peaks shift toward lower energies when H_2_ is introduced (Figure 5b). The PDOS analysis reveals clear hybridization between the H_2_ and the Ca atom (Figure 5d), mainly within −9.983 eV to −8.632 eV and −0.032 eV to +6.873 eV. Additionally, the TED plots in Figure 6c show marked overlap of the H_2_ molecule with the 1Ca-silicene surface, reinforcing the presence of a strong interaction [42]. According to EDD and Mulliken analyses, the charge transfer from pristine silicene to H_2_ is merely 0.02 |e| (Figure 6b), whereas Ca decoration raises this value markedly to 0.16 |e|, as seen in the EDD slice of Figure 6d. To cross-check the Mulliken charges, Hirshfeld analysis was also conducted for the 1H-1Ca-silicene system [54]. The Hirshfeld method gives a charge transfer of roughly 0.1 |e| from the substrate to the H_2_ molecule, which agrees qualitatively with the Mulliken value of 0.16 |e|. The discrepancy in absolute magnitudes between the two schemes is unsurprising, given their different theoretical underpinnings. Importantly, both approaches consistently show that Ca donates electrons while H_2_ accepts them. This consistency reveals that the charge-transfer trends reported here are genuine and not an artifact of the Mulliken partitioning. With this charge-transfer picture confirmed, the adsorbed H_2_ molecule becomes polarized on the 1Ca-silicene surface. The polarization manifests as electron accumulation near the Ca site and electron depletion on the far side of the molecule, with these two regions distributed on opposite sides of the H_2_ [4]. Charge accumulation near the Ca site, appearing as a red region in the EDD map (Figure 6d), corresponds to the σ* orbital of H_2_, while charge depletion on the far side, shown in blue, corresponds to the σ orbital [48]. This pattern indicates that electrons are first transferred from the σ orbital of H_2_ to the empty *d* states of Ca, followed by back-donation from Ca into the σ* orbital of H_2_. The H_2_ molecule is therefore captured by 1Ca-silicene through a Kubas-like interaction [44,55].

### 3.3. Hydrogen Storage Performance of Ca-Decorated Silicene

**Figure 7:**
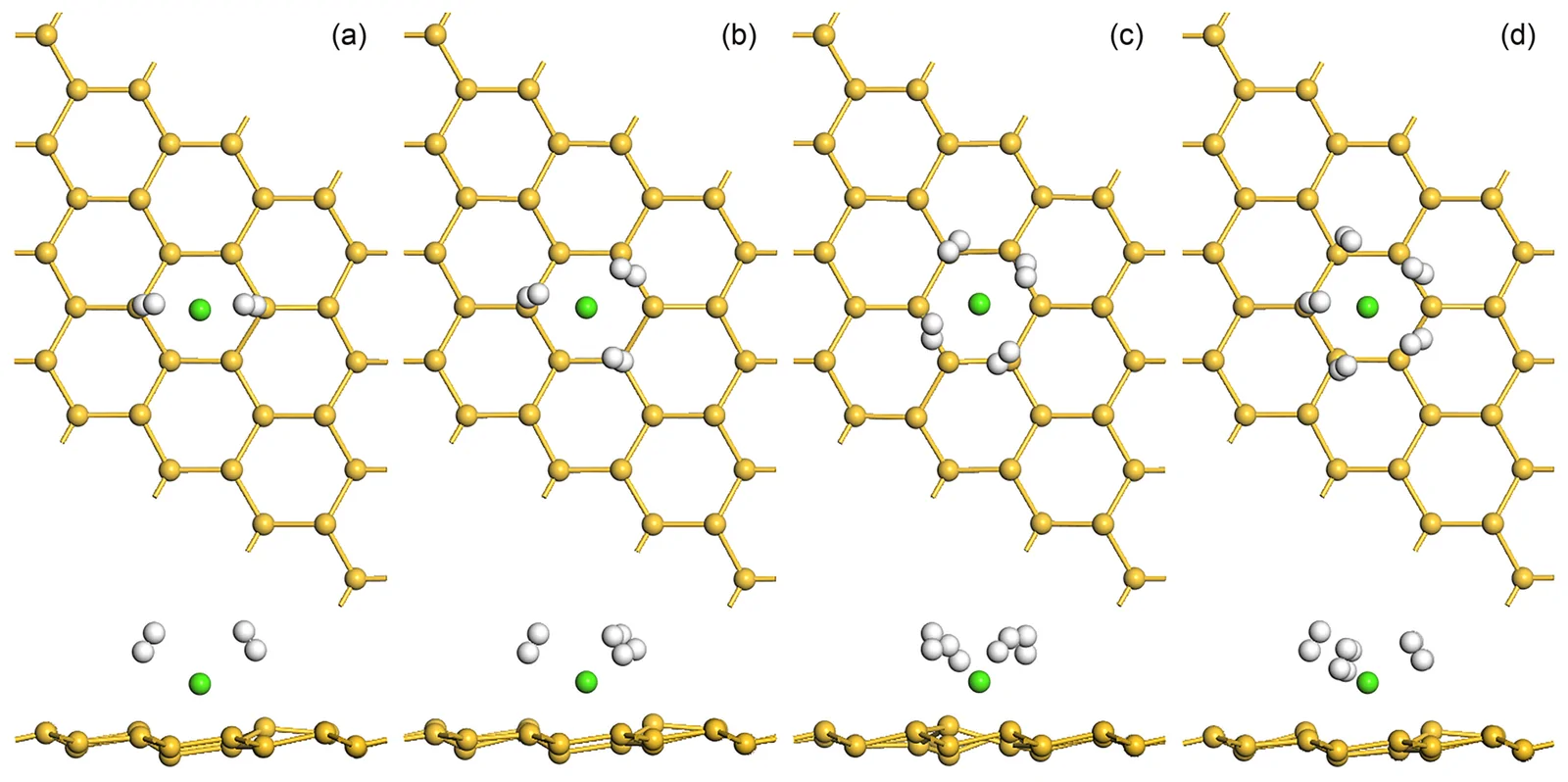
Top and side views of (**a**) two, (**b**) three, (**c**) four, and (**d**) five H_2_ molecules attached to 1Ca-silicene.

To probe the maximum H_2_ uptake capacity of 1Ca-silicene, we successively introduced additional H_2_ molecules to the system. The resulting optimized configurations, shown in Figure 7, demonstrate that two to five H_2_ molecules can be stably accommodated by 1Ca-silicene. These systems are denoted as 2H-1Ca-silicene through 5H-1Ca-silicene, respectively. The *E_ads_*_−*av*_ decreases progressively with increasing H_2_ loading, from −0.504 eV for two H_2_ to −0.327 eV for five H_2_. The *E_ads_*_−*n*_ for the *n*-th H_2_ follow a similar declining trend, with values of −0.475 eV, −0.406 eV, −0.352 eV, and −0.284 eV for the second through fifth H_2_, respectively. When introducing more than five H_2_ molecules, the additional ones move away from the Ca site, suggesting that the saturation capacity of a single Ca atom on silicene is limited to five H_2_ molecules. To confirm this limit, we attempted to introduce a sixth H_2_ molecule to the 5H-1Ca-silicene system, with an initial Ca-H_2_ distance of 2.0 Å, a distance commonly adopted in similar adsorption studies [7,56]. After full relaxation, as displayed in Figure S3, the sixth H_2_ molecule drifted away from the Ca site to a final distance of 3.804 Å, whereas the other five H_2_ molecules maintained configurations resembling those in the 5H-1Ca-silicene structure. The adsorption energy for the sixth H_2_ molecule is only −0.018 eV, indicating a negligible interaction with the substrate [56]. These observations indicate that the sixth H_2_ cannot be effectively stored on the 1Ca-silicene surface, revealing that five H_2_ molecules per Ca atom represent the true saturation capacity.

**Figure 8:**
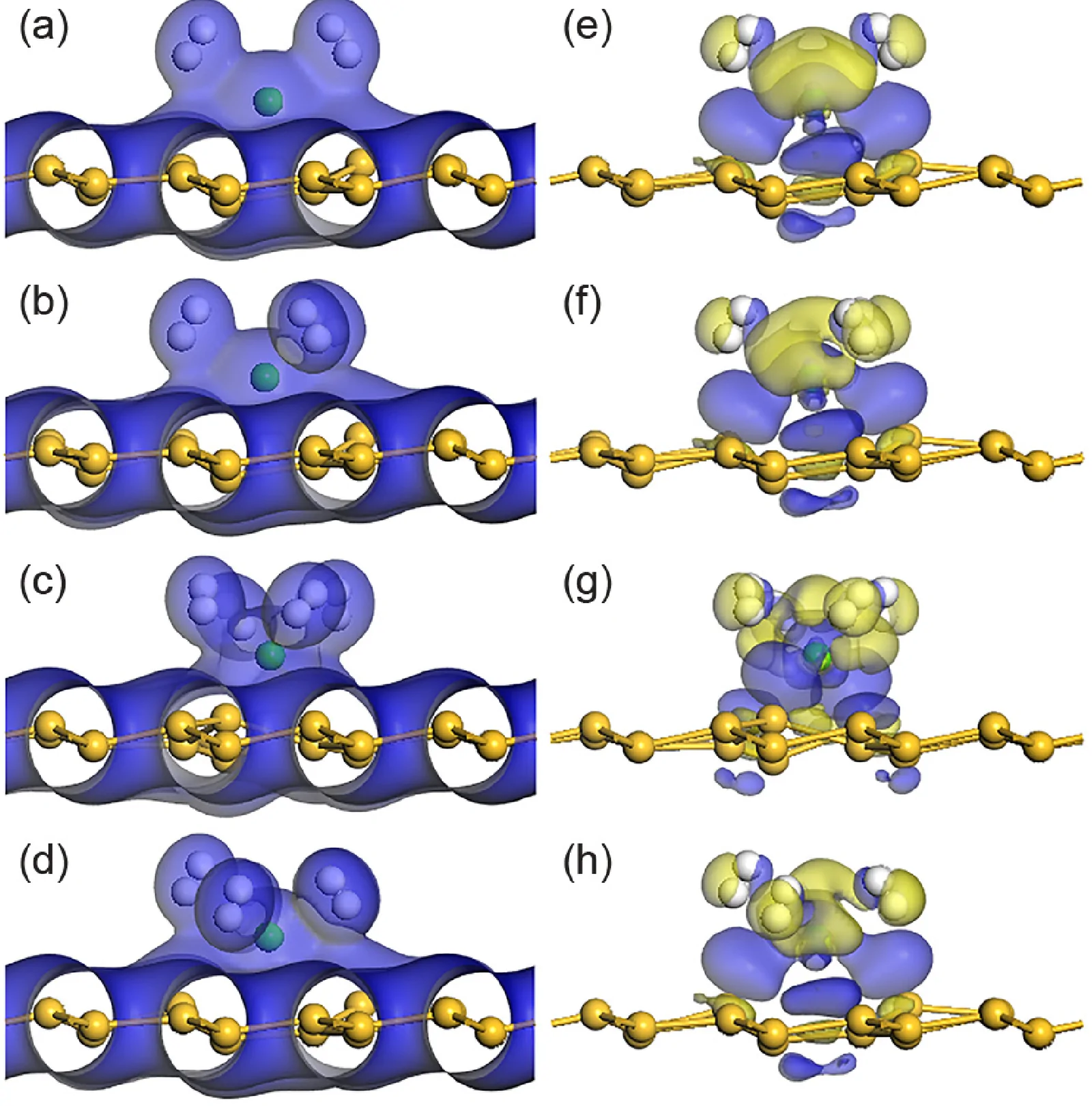
TED and EDD distributions for (**a**,**e**) two, (**b**,**f**) three, (**c**,**g**) four, and (**d**,**h**) five H_2_ molecules on 1Ca-silicene. The isosurface value for the EDD maps (**e**–**h**) is set to 0.015 e/Å^3^, with charge accumulation and depletion represented by blue and yellow, respectively.

**Figure 9:**
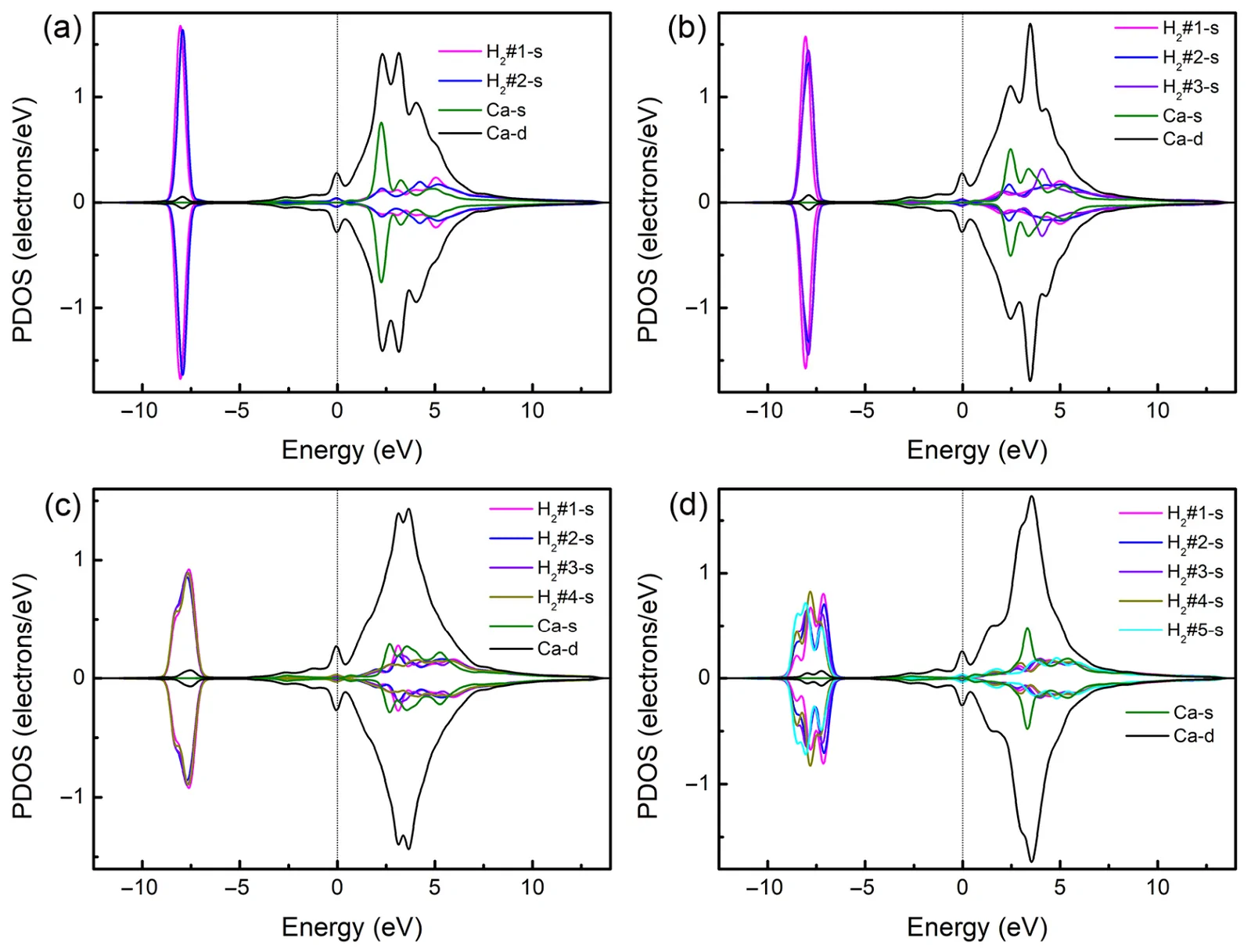
PDOS for (**a**) two, (**b**) three, (**c**) four, and (**d**) five H_2_ molecules on 1Ca-silicene.

To better elucidate the hydrogen storage performance of 1Ca-silicene, PDOS, EDD, and TED analyses were performed for each of the multi-H_2_ configurations. The TED plots in Figure 8a–d reveal pronounced and clear overlap between the adsorbed H_2_ molecules and the Ca-decorated substrate, pointing to strong interactions across all loading levels [57]. This is further supported by the PDOS data in Figure 9, which reveal strong mixing between the H_2_ *s* states and the Ca *s*/*d* states for the 2H through 5H systems [44]. Specifically, for the 5H-1Ca-silicene system, the *s* states of the five H_2_ molecules exhibit extensive hybridization with the Ca *s*/*d* states over two distinct energy intervals: −9.273 eV to −6.332 eV and −3.178 eV to +6.785 eV (Figure 9d). The EDD analysis in Figure 8e–h reveals that the adsorbed H_2_ molecules act as charge acceptors on the 1Ca-silicene surface. For the five-H_2_ configuration, each H_2_ molecule receives 0.11 |e|, 0.11 |e|, 0.10 |e|, 0.10 |e|, and 0.11 |e|, respectively, giving a total of 0.53 |e| transferred from the 1Ca-silicene to the H_2_ molecules. This substantial charge transfer generates a strong electrostatic attraction between the positively polarized Ca site and the negatively charged H_2_ molecules, which effectively stabilizes the multi-H_2_ adsorption configurations [10,48,58]. The corresponding EDD slice (Figure S5), taken from the perspective view shown in Figure S4, displays a charge accumulation region near the Ca and a depletion zone on the outer side of each H_2_. This pattern is consistent with the σ-donation and *d*-σ* back-donation pathway observed for single H_2_ adsorption (Figure 6d), confirming that the five H_2_ molecules are captured through the same Kubas-like interaction [55].

### 3.4. Hydrogen Release Performance of Ca-Decorated Silicene with Two-Side Ca Decoration

**Figure 10:**
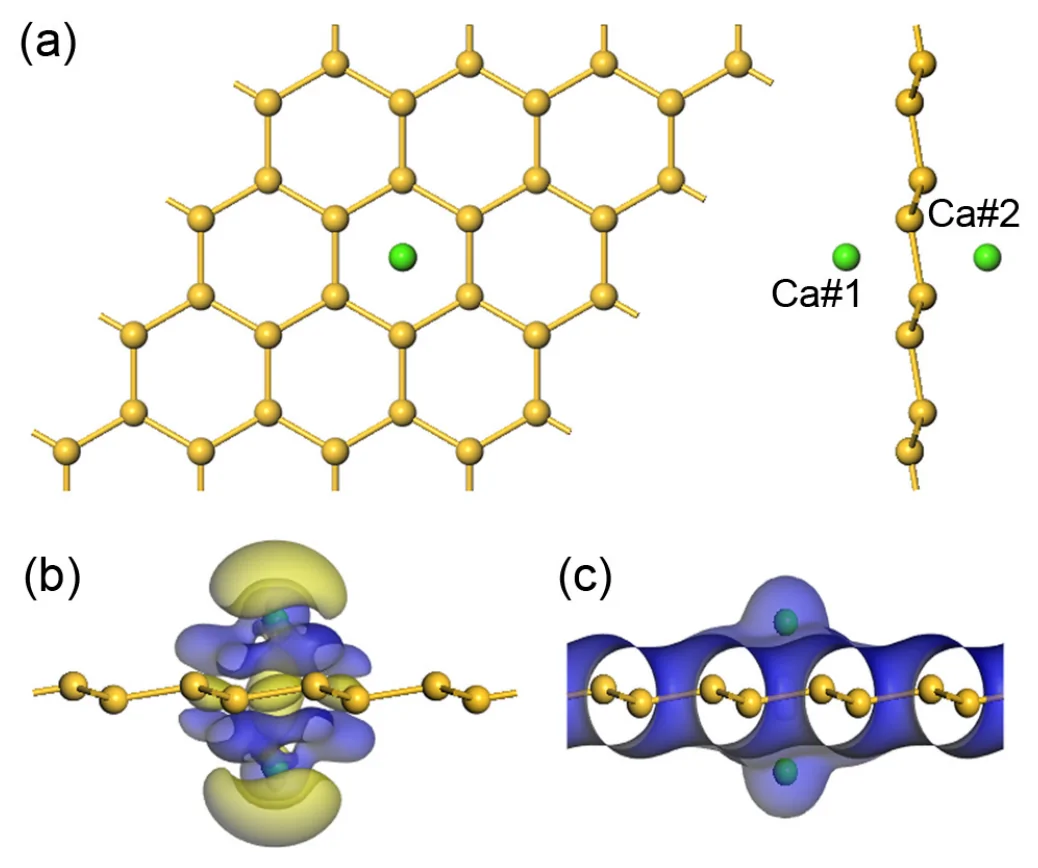
Optimized geometry of two Ca atoms placed on opposite sides of the silicene monolayer: (**a**) top/side views, (**b**) EDD isosurface map, and (**c**) TED. The isosurface value is set to 0.015 e/Å^3^ in (**b**), with charge accumulation and depletion represented by blue and yellow, respectively.

**Figure 11:**
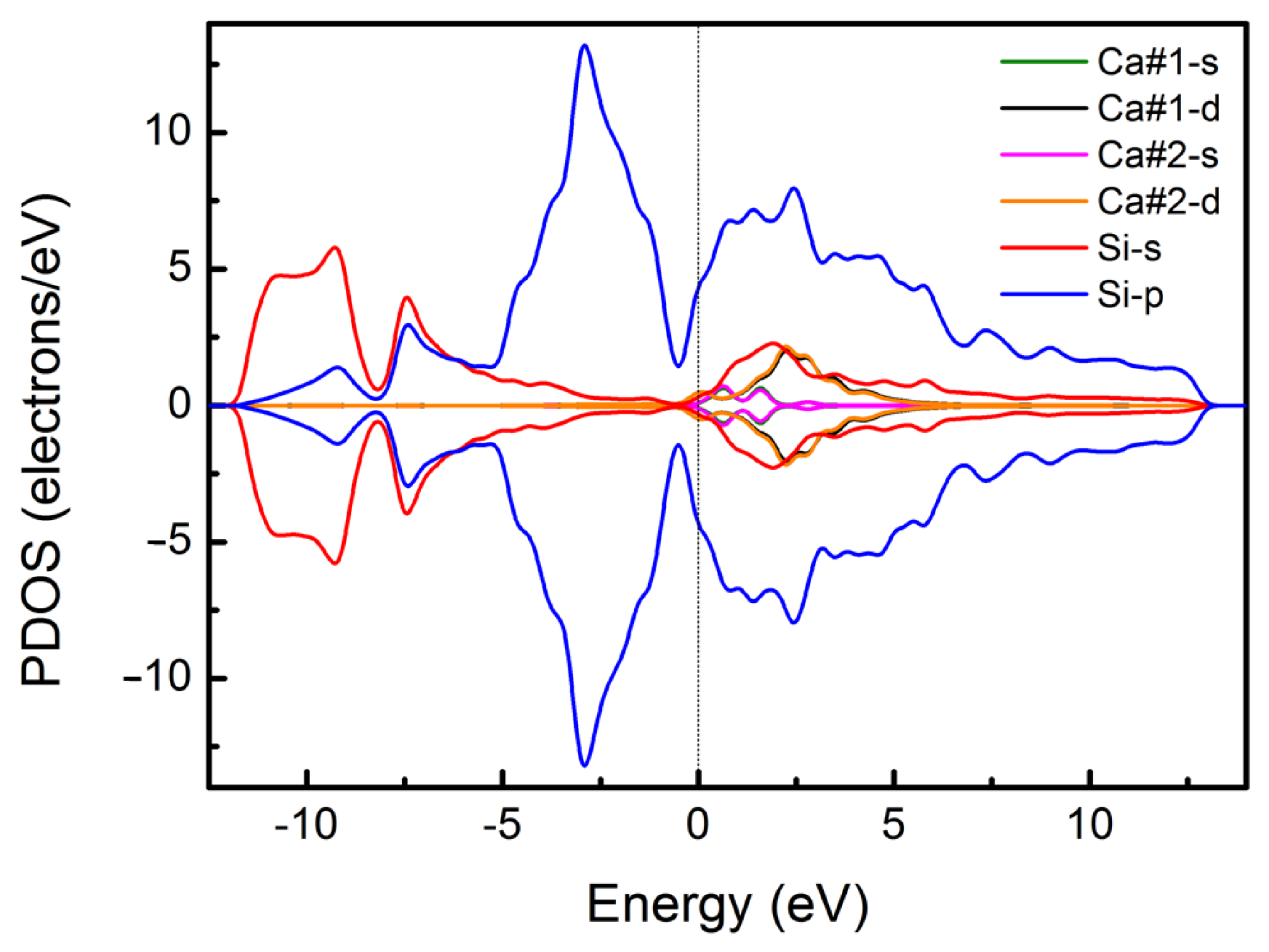
PDOS for two Ca atoms adsorbed on opposite sides of the silicene sheet.

Based on the findings from the single-Ca system, we extended the investigation to a configuration with two Ca atoms placed on opposite sides of the silicene monolayer, denoted as 2Ca-silicene (Figure 10a). Both Ca atoms remain firmly attached to the surface, with an average binding energy of −2.194 eV, which is greater than the bulk Ca cohesive energy (1.84 eV), confirming the energetic feasibility of double-sided Ca decoration [44]. The two Ca atoms are positioned 1.895 Å and 1.898 Å away from the silicene plane on opposite sides, respectively, comparable to that observed for the single Ca system (1.816 Å). To further verify the dynamical stability of the double-sided Ca decoration, we calculated the phonon spectrum of the 2Ca-silicene configuration. As shown in Figure S6, the phonon dispersion curve exhibits no imaginary frequencies throughout the entire Brillouin zone, implying that the 2Ca-silicene system is dynamically stable [43]. PDOS results in Figure 11 exhibit pronounced orbital coupling between the two Ca atoms and the silicene within the energy window of −0.580 eV to +3.174 eV, indicating robust electronic coupling [59]. The pronounced overlap seen in the TED plots (Figure 10c) lends additional support to this conclusion. Charge redistribution occurs upon Ca decoration: the Ca atoms on the upper and lower sides donate 1.19 |e| and 1.18 |e|, respectively (Figure 10b), generating a strong electrostatic attraction that stabilizes the 2Ca-silicene configuration [51,52].

**Figure 12:**
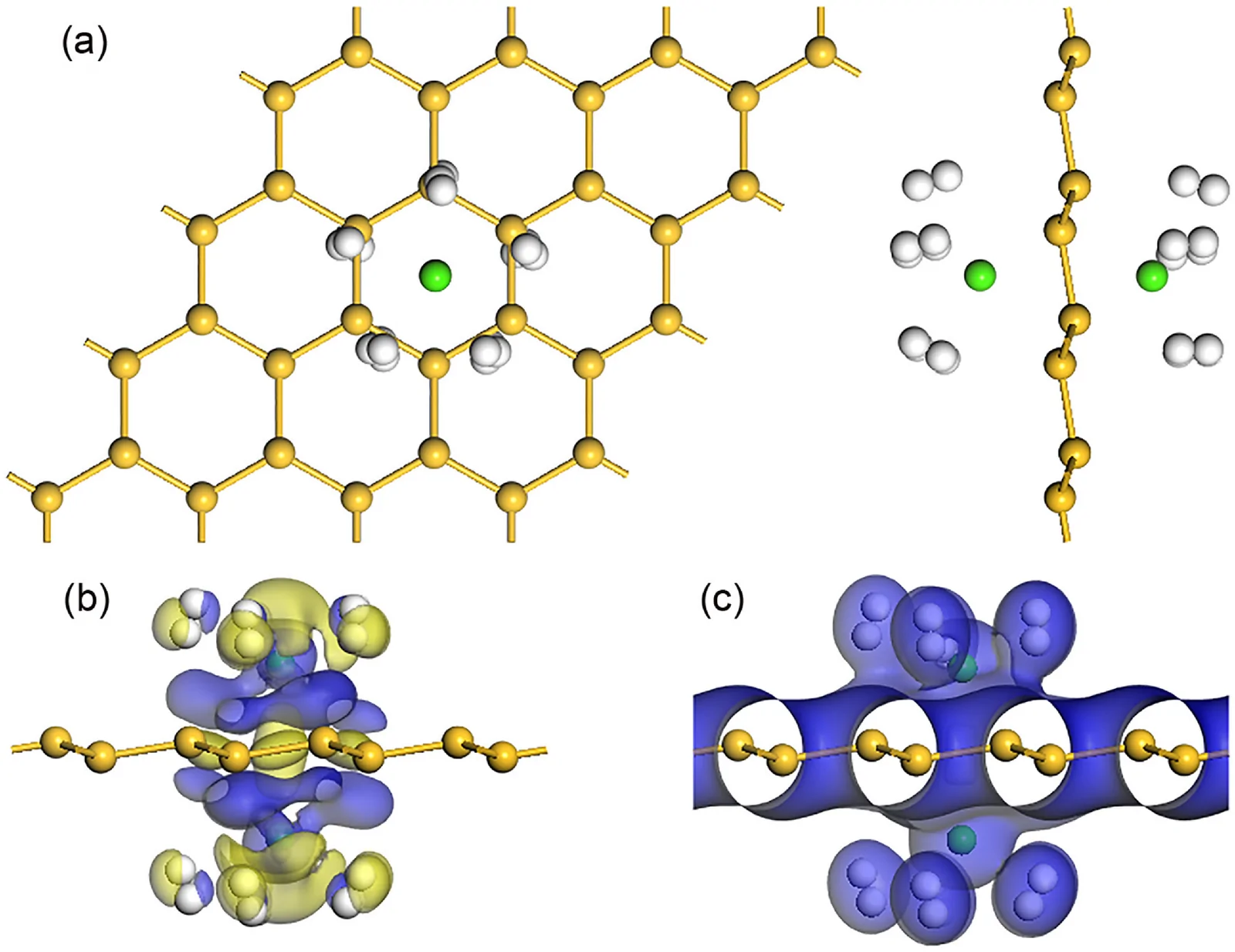
Optimized structure of ten H_2_ molecules stored on two Ca atoms anchored on opposite sides: (**a**) top/side views, (**b**) EDD, and (**c**) TED. The isosurface value is set to 0.015 e/Å^3^ in (**b**), with charge accumulation and depletion represented by blue and yellow, respectively.

We next examined the H_2_ uptake performance of 2Ca-silicene by introducing ten H_2_ molecules. As shown in Figure 12a, five H_2_ molecules remain bound to each Ca atom, adopting configurations similar to those in the 5H-1Ca-silicene. The Ca-H_2_ separations fall between 2.514 Å and 2.545 Å, comparable to the single-Ca values. For the ten-H_2_ system, the *E_ads_*_−*av*_ is −0.304 eV, slightly lower than that for five H_2_ on 1Ca-silicene. The H-H bond lengths of the ten H_2_ are stretched to values between 0.765 Å and 0.769 Å, exceeding the free H_2_ bond length and pointing to notable bond activation [50]. These observations indicate that 2Ca-silicene effectively binds all ten H_2_ molecules. The TED plots in Figure 12c show substantial overlap between the five H_2_ molecules and their respective Ca adsorption sites, further confirming strong interactions. Charge transfer analysis (Figure 12b) indicates that the H_2_ molecules on the upper and lower Ca atoms capture 0.49 |e| and 0.48 |e|, respectively, from the 2Ca-silicene. These findings indicate that the 2Ca-silicene system can stably store ten H_2_ with an average adsorption energy of −0.304 eV, within the DOE window [52,60]. Furthermore, the configuration, with two Ca atoms anchored on opposite sides of a six-membered Si ring and ten H_2_ molecules accommodated, delivers a hydrogen storage capacity of 7.5 wt%, exceeding the DOE benchmark [33]. To better assess the performance of the present system, Table 2 presents a comparison of the hydrogen storage properties of various metal-decorated silicene and Ca-decorated 2D materials reported in the literature. The capacity obtained in this study (7.5 wt%) exceeds most previously reported values, while the average adsorption energy (−0.304 eV) falls in the favorable range for reversible storage. These comparisons suggest that Ca-decorated silicene offers competitive advantages for hydrogen storage applications.

**Figure 13:**
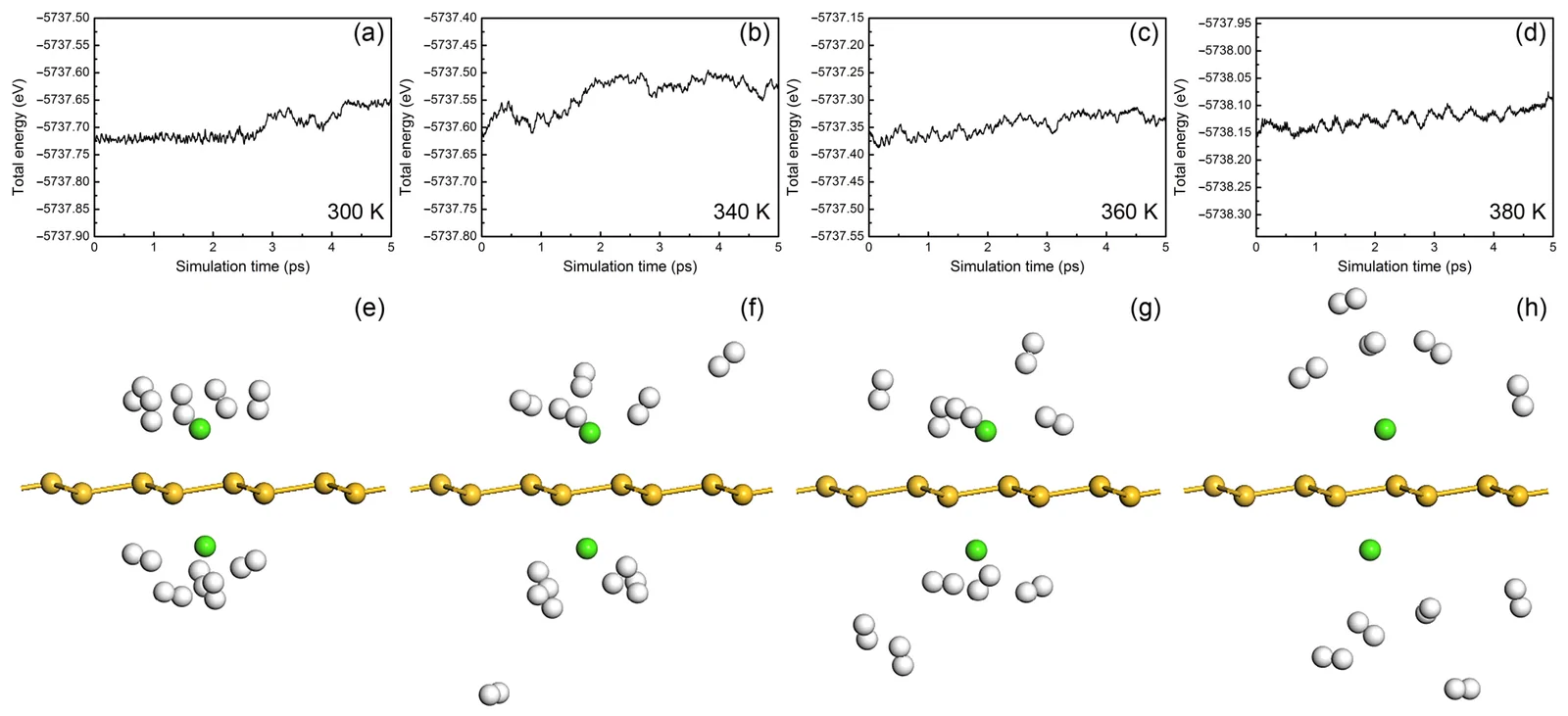
Frames extracted from AIMD simulations illustrating the thermal release of ten H_2_ from 2Ca-silicene at (**a**,**e**) 300 K, (**b**,**f**) 340 K, (**c**,**g**) 360 K, and (**d**,**h**) 380 K.

The desorption temperature (*T_d_*) was derived from the Van ’t Hoff relation outlined in the Computational details section (Equation (4)) [65,66]. Using the *E_ads_*_−*av*_ of −0.304 eV for the ten H_2_ molecules on 2Ca-silicene, the calculated desorption temperature is roughly 391 K. We also conducted NVT ensemble AIMD simulations using the Nosé–Hoover thermostat to investigate the release characteristics [31]. These simulations were performed over the temperature interval of 300–380 K, with each run lasting 5 ps at a time step of 1.0 fs, during which the trajectories of the adsorbed H_2_ molecules were closely followed. At 300 K, all ten H_2_ molecules stay attached to the 2Ca-silicene surface (Figure 13a,e), with configurations similar to those at 0 K (Figure 12a). Upon raising the temperature to 340 K (Figure 13b,f), one H_2_ molecule leaves each Ca site, while the other four remain bound. At 360 K (Figure 13c,g), three H_2_ molecules detach from the upper Ca center and two from the lower one. At 380 K (Figure 13d,h), the surface becomes completely free of H_2_, and the Ca-decorated silicene structure undergoes no appreciable distortion over the course of the simulation. These observations confirm that complete release of the stored H_2_ from Ca-decorated silicene occurs at approximately 380 K, with both the substrate and the Ca adatoms retaining their structural integrity.

Our calculations show that each Ca site binds up to five H_2_ molecules, and double-sided Ca coverage doubles the overall capacity. The resulting system achieves 7.5 wt% storage with an average adsorption energy of −0.304 eV, satisfying the U.S. DOE criterion. It should be emphasized that the present calculations were performed on idealized free-standing silicene models. Under realistic conditions, several factors may affect the predicted hydrogen storage performance. For example, silicene is reactive in ambient environments, and surface oxidation could reduce the number of active Ca sites or alter the electronic properties of the substrate [67]. Substrate support, such as Ag(111), which is commonly employed for silicene growth, might also affect Ca binding and subsequent H_2_ adsorption [68]. Intrinsic defects, including vacancies and Stone–Wales defects, could modify the local charge distribution and the interaction strength with Ca and H_2_ [69]. Finite Ca coverage, in contrast to the idealized periodic arrangement assumed here, might influence the uniformity of adatom dispersion and the overall storage capacity [70,71]. Despite these caveats, the present study offers atomic-level understanding of the Ca-silicene-H_2_ interaction and establishes a theoretical basis for future investigations that incorporate such practical factors. Overall, our results suggest that Ca-decorated silicene represents a potential material for reversible hydrogen storage.

## 4. Conclusions

In conclusion, we have performed a systematic DFT investigation of H_2_ adsorption on Ca-functionalized silicene. The hollow site of the silicene monolayer is identified as the most favorable position for single Ca atom adsorption, with a binding energy of −2.348 eV, whose magnitude is larger than the cohesive energy of bulk Ca (1.84 eV). Such a strong anchoring interaction prevents Ca aggregation and promotes uniform dispersion of adatoms on the surface. H_2_ binds weakly to pristine silicene, with an adsorption energy of merely −0.047 eV. Introducing Ca atoms considerably increases the H_2_ adsorption energy to −0.561 eV, reflecting a marked improvement in binding strength. Each Ca site can capture a maximum of five H_2_ molecules, with average adsorption energies varying between −0.504 eV and −0.327 eV. Placing Ca atoms on both faces of the silicene sheet raises the total H_2_ uptake to ten molecules, with an average adsorption energy of −0.304 eV. This double-sided configuration reaches a storage capacity of 7.5 wt%, which surpasses the DOE benchmark. Complete desorption of the stored H_2_ occurs at about 380 K, while the Ca-decorated silicene structure remains intact. These theoretical findings reveal the potential of Ca-decorated silicene as a reversible hydrogen storage material.

## Figures and Tables

**Table 1:** Calculated binding energy (*E_b_*_−*Ca*_), vertical height (*H_Ca_*_−*silicene*_) between the Ca atom and the silicene plane, and charge transfer (*Q_Ca_*_−*silicene*_) from Mulliken analysis for Ca decoration at the three high-symmetry sites.

Site	*E_b_*_−*Ca*_ (eV)	*H_Ca_*_−*silicene*_ (Å)	*Q_Ca_*_−*silicene*_ (|e|)
T	−1.729	2.137	0.62
B	−1.865	2.091	0.74
H	−2.348	1.816	1.16

**Table 2:** Comparison of hydrogen storage performance for metal-decorated silicene and Ca-decorated 2D materials.

Material	Metal Species	Average Adsorption Energy (eV/H_2_)	Hydrogen Storage Capacity (wt%)	Desorption Temperature (K)	Ref.
Silicene	Li	−0.17	6.35	217.5	[25]
Silicene	Ni	−0.439	0.21	561.7	[26]
Silicene	Li-Na	−0.29	6.65	371.1	[41]
Silicene	V	−0.97	~4.8	~1241.1	[61]
Silicene	Be	−0.69	~1.5	~882.8	[61]
Silicene	Au	~−0.12	~3.1	~153.5	[61]
Silicene	Ti	~−0.51	~4.9	~652.5	[61]
Defective graphene	Ca	−0.33	5.2	422.2	[28]
BN nanosheets with B vacancies	Ca	~−0.20	7.6	~255.9	[29]
Irida-graphene	Ca	−0.177	8.0	226.5	[31]
B_4_CN_3_ monolayer	Ca	−0.25	5.51	319.8	[49]
Biphenylene	Ca	~−0.30	~6.8	~383	[55]
ψ-graphene	Ca	~−0.30	~4.2	~383	[55]
2D carbon allotropes	Ca	−0.1 to −0.24	3.53 to 4.48	127.9 to 307.1	[62]
Borophene	Ca	−0.23	7.2	294.3	[63]
Boron-doped BlueP monolayer	Ca	−0.17	7.29	217.5	[64]
Silicene	Ca	−0.304	7.5	~380	This work

## Data Availability

The original contributions presented in this study are included in the article/Supplementary Material. Further inquiries can be directed to the corresponding authors.

## Supplementary Materials

The following supporting information can be downloaded at: https://www.mdpi.com/article/10.3390/nano16171060/s1, Figure S1: Diffusion energy barrier for a single Ca atom migrating from one hollow site to an adjacent hollow site on the silicene surface, with a calculated barrier of 1.451 eV. The initial state (IS), transition state (TS), and final state (FS) configurations are also shown in the figure; Figure S2: Calculated phonon dispersion of one H_2_ adsorbed on 1Ca-silicene; Figure S3: (a) Initial structure and (b) optimized structure of the six H_2_ molecules attached to 1Ca-silicene; Figure S4: Top view (a) and perspective view (b) of the optimized silicene structure. The view in (b) corresponds to a rotation of (a) by approximately 45^o^ toward the page plane; Figure S5: EDD slice maps for five H_2_ adsorbed on 1Ca-silicene, taken from the perspective view shown in Figure S4. The color scale ranges from −0.05 to +0.05 e/Å^3^, with red indicating charge accumulation and blue indicating charge depletion; Figure S6: Phonon dispersion curves for the 2Ca-silicene configuration.
