# Supplementary material for: High-Capacity Reversible Hydrogen Storage on Calcium-Decorated Silicene: A DFT Study
Source: Nanomaterials (Basel). 2026 Aug 26;16(17):1060. doi: 10.3390/nano16171060 (PMC13567285; doi:10.3390/nano16171060)
Supplement: Supplementary file 1 [file nanomaterials-16-01060-s001.zip › nanomaterials-4492857-supplementary.pdf]

High-Capacity Reversible Hydrogen Storage on Calcium-Decorated Silicene: A DFT Study

Na Xiong [1,†], Ying Zhang [1,†], Lun Tan [1,2], Gui Lei [1], Shulin Yang [1,*] and Zhigao Lan [1,*]

[1] School of Physics and Electronic Information, Huanggang Normal University, Huanggang 438000, China
[2] Faculty of Physics and Electronic Sciences, Hubei University, Wuhan 430062, China
* Correspondence: yangsl@hgnu.edu.cn (S.Y.); lanzhg@hgnu.edu.cn (Z.L.)
† These authors contributed equally to this work.

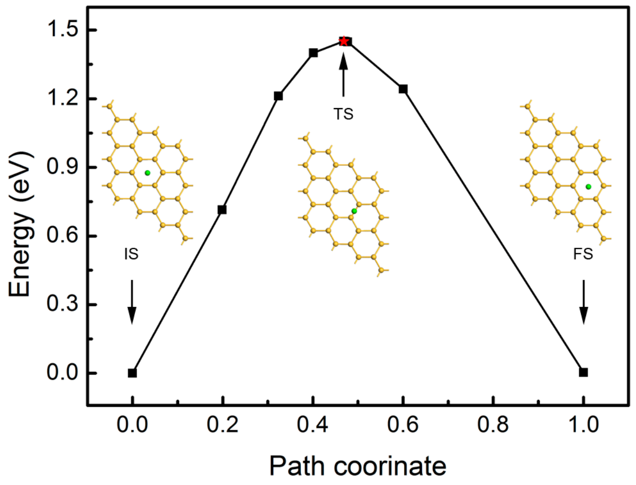

Figure S1 Diffusion energy barrier for a single Ca atom migrating from one hollow site to an adjacent hollow site on the silicene surface, with a calculated barrier of 1.451 eV. The initial state (IS), transition state (TS), and final state (FS) configurations are also shown in the figure.

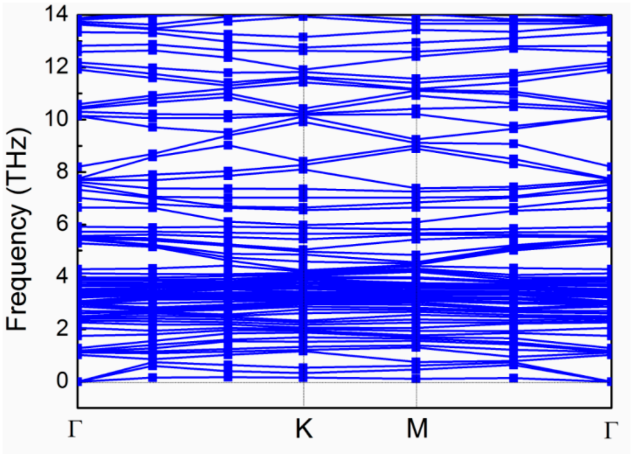

Figure S2 Calculated phonon dispersion of one H_2 adsorbed on 1Ca-silicene.

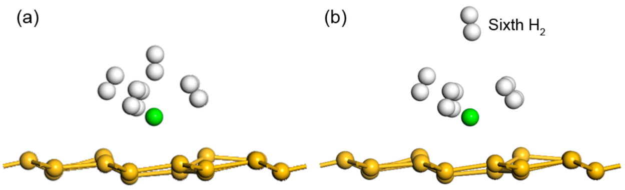

Figure S3 (a) Initial structure and (b) optimized structure of the six H_2 molecules attached to 1Ca-silicene.

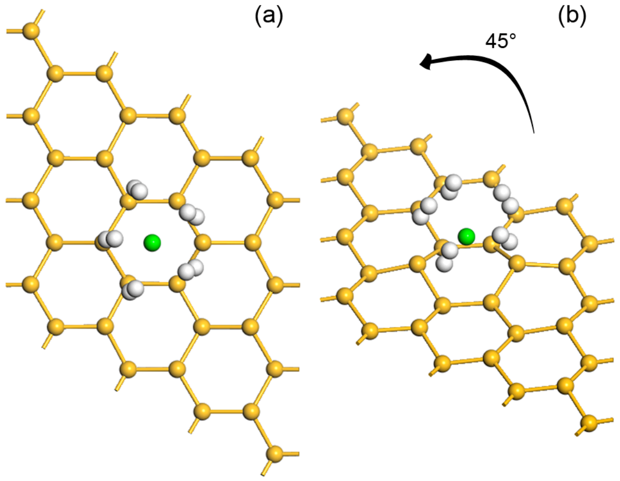

Figure S4 Top view (a) and perspective view (b) of the optimized silicene structure. The view in (b) corresponds to a rotation of (a) by approximately 45° toward the page plane.

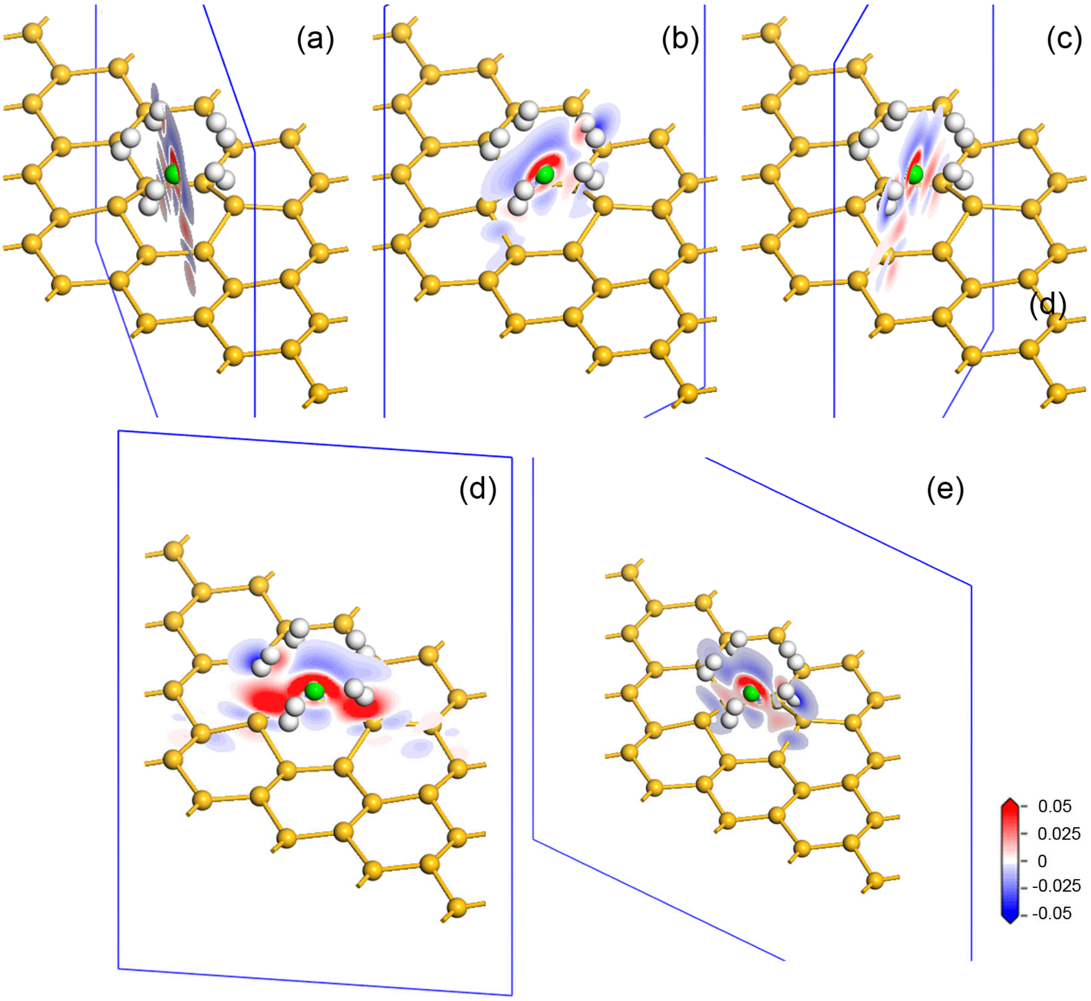

Figure S5 EDD slice maps for five H_2 adsorbed on 1Ca-silicene, taken from the perspective view shown in **Figure S4**. The color scale ranges from -0.05 to +0.05 e/Å^3, with red indicating charge accumulation and blue indicating charge depletion.

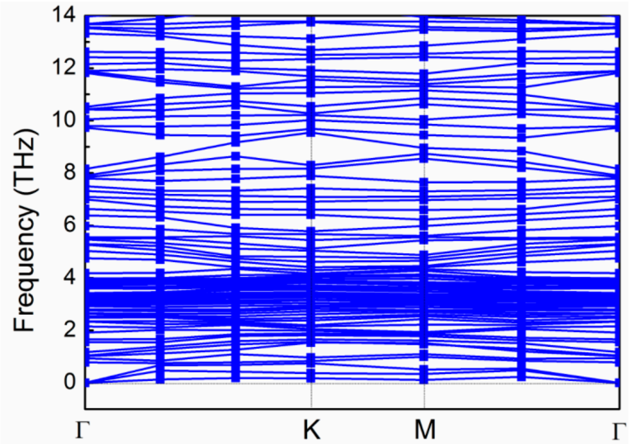

Figure S6 Phonon dispersion curves for the 2Ca-silicene configuration.
